# Quantitative comparison of correction techniques for removing systemic physiological signal in functional near-infrared spectroscopy studies

**DOI:** 10.1117/1.NPh.7.3.035009

**Published:** 2020-09-23

**Authors:** Hendrik Santosa, Xuetong Zhai, Frank Fishburn, Patrick J. Sparto, Theodore J. Huppert

**Affiliations:** a University of Pittsburgh, Department of Radiology, Pittsburgh, Pennsylvania, United States; b University of Pittsburgh, Department of Bioengineering, Pittsburgh, Pennsylvania, United States; c University of Pittsburgh, Department of Psychiatry, Pittsburgh, Pennsylvania, United States; d University of Pittsburgh, Department of Physical Therapy, Pittsburgh, Pennsylvania, United States; e University of Pittsburgh, Clinical Science Translational Institute, Pittsburgh, Pennsylvania, United States; f University of Pittsburgh, Center for the Neural Basis of Cognition, Pittsburgh, Pennsylvania, United States; g University of Pittsburgh, Department of Electrical and Computer Engineering, Pittsburgh, Pennsylvania, United States

**Keywords:** functional near-infrared spectroscopy, systemic physiological noise, noise removal, short-separation measurements, sensitivity–specificity

## Abstract

**Significance:** Isolating task-evoked brain signals from background physiological noise (e.g., cardiac, respiratory, and blood pressure fluctuations) poses a major challenge for the analysis of functional near-infrared spectroscopy (fNIRS) data.

**Aim:** The performance of several analytic methods to separate background physiological noise from brain activity including spatial and temporal filtering, regression, component analysis, and the use of short-separation (SS) measurements were quantitatively compared.

**Approach:** Using experimentally recorded background signals (breath-hold task), receiver operating characteristics simulations were performed by adding various levels of additive synthetic “brain” responses in order to examine the sensitivity and specificity of several previously proposed analytic approaches.

**Results:** We found that the use of SS fNIRS channels as regressors of no-interest within a linear regression model was the best performing approach examined. Furthermore, we found that the addition of all available SS data, including all recorded channels and both hemoglobin species, improved the method performance despite the additional degrees-of-freedom of the models. When SS data were not available, we found that principal component filtering using a separate baseline scan was the best alternative.

**Conclusions:** The use of multiple SS measurements as regressors of no interest implemented in a robust, iteratively prewhitened, general linear model has the best performance of the tested existing methods.

## 1 Introduction

Functional near-infrared spectroscopy (fNIRS) is a neuroimaging technique that provides the opportunity to noninvasively monitor hemodynamic activity within the human brain. First demonstrated by Jobsis,^1^ the fNIRS technique has been applied to a growing number of populations and experimental studies over the last three decades (reviewed in Refs. 2 and 3). This technique uses diffuse optical measurements in the red to near-infrared range (650 to 900 nm) to measure changes in cerebral blood oxygenation and concentration. These signals are recorded between optical light sources and detectors that are placed on the scalp over the region-of-interest. At the typical source–detector separations used for functional brain imaging of around 25 to 35 mm, light from the scalp can penetrate into the first few millimeters of the cerebral tissue, which allows measurements of many cortical cognitive areas of the brain.^2^ However, since these measurements are made across layers of the highly vascularized skin and skull, fNIRS measurements are often heavily contaminated by these superficial physiological signals (e.g., blood pressure, respiratory, and cardiac signals).^4^^,^^5^ These superficial systemic physiological noises can be misinterpreted as brain activity and can drive both high false positive and false negative estimates.^6^ Furthermore, these errors are particularly troublesome when there is a possibility of a task-induced systemic change accompanying the evoked brain signals, as may be the case in studies requiring physical movement or inducing anxiety resulting in respiratory and heart rate changes.^5^

Over the last two decades, there have been numerous proposed methods for addressing systemic contamination in fNIRS measurements (see Table 1 for a summary of published methods). In general, these methods can be categorized into either prefiltering or statistical approaches. Prefiltering methods use a two-step process to first attempt to first remove or correct the noise prior to running a statistical analysis model. For example, an algorithm could be applied to remove the artifacts from raw data generating a filtered dataset. After filtering, a block averaging or regression model is then applied to the filtered dataset to estimate the evoked hemodynamic signals. In this case, any statistics on the estimates of brain activity are inherently conditional on preprocessing steps.^28^ Aggressive prefiltering may reduce systemic contamination but potentially lower sensitivity and introduce additional type-II statistical errors (false negatives).^5^ Examples of prefiltering methods include bandpass filter,^11^^,^^12^ principal component analysis (PCA)/independent component analysis (ICA),^14^^,^^19^ adaptive filtering methods,^7^ and prefilters designed from short-separation (SS) fNIRS measurements.^13^^,^^21^^–^^24^ A detailed review of the prefiltering corrections is provided by Pinti et al.^28^

**Table 1:** Survey of different processings to remove systemic noise in fNIRS signal

Processing	Citation
Adaptive filter	7
AR-IRLS^a^^,^^b^	8 and 9
bPCA^a^	10
Bandpass filter^a^	11 and 12
GLM using SS filter^a^	13
ICA	11, 14, and 15
ICA using SS channels	16
Kalman filter	17 and 18
PCA^a^	19 and 20
SS as a regressor^a^^,^^b^	13 and 21–24
OLS^a^^,^^b^	25
Wavelet analysis	26 and 27

a Method implemented in this current work for comparison.

b Statistical approach.

An alternative to the two-step prefiltering/statistical analysis approach (see “b” in Table 1) is to integrate the corrections directly into the statistical model in a single step. When using a statistical approach for accounting for systemic noise, a model of the brain activity is generalized to account for systemic physiology. The most common statistical method involves the addition of regressors of no interest into a linear regression model to estimate task-related activity. For example, these nuisance regressors could be external measurements of physiology (e.g., finger pulse oximeter or a respiratory belt)^12^^,^^17^ or estimates of the scalp response from SS fNIRS measurements.^13^ However, a challenge of these regressor models is collinearity introduced between the task and nuisance regressors, which can happen if the systemic physiological response is correlated with the performance of the task. Collinearity in the regression analysis can destabilize it due to poor mathematical conditioning of the model and can produce unpredictable results.

Alternatively, modifications to the statistical assumptions of the model itself can be made through precoloring^26^ or prewhitening^8^^,^^9^ approaches. In both prewhitening and precoloring, the linear model is generalized to correct errors due to the incorrect statistical assumptions that physiological noise is uncorrelated, normally distributed and white (cf., structured or colored noise). Precoloring and prewhitening methods, however, do not account for errors due to the nonstationarity of the noise or its potential to be synchronized to the task (e.g., heart rate increases during a walking task).^5^ These statistical corrections are not exclusive to the use of nuisance regressors in the model or additional preprocessing stages and all approaches can be used together creating a large array of possible analysis pipelines.^5^

The objective of this study was to compare the sensitivity and specificity of various previously published methods including the use of SS measurements. In this work, we compared many possible combinations of various pipelines with/without preprocessing of PCA, SS-filter with several different regression models [i.e., ordinary least-squares (OLS), iterative autoregressive least-squares (AR-IRLS), and mixed-effects (ME) version]. We also investigated the use of SS measurements as regression for solving general linear model (GLM). These various analysis pipelines were quantitatively compared using receiver operating characteristic (ROC) analysis using semisynthetic simulations^29^^,^^30^ (e.g., real experimental physiological data from a resting state (RS) scan and a purposeful breath-hold (BH) task with known artificial “brain activity signals” added to the simulations). In addition, we compared the performance of the models based on the number of SS channels (from only the nearest one to all eight channels). Based on the area under the curve (AUC) of the ROC comparisons, we found that the use of SS measurements as regressors using AR-IRLS for solving GLM has the best performance of the tested existing methods.

## 2 Theory

In Table 1, we summarize several studies that describe approaches to dealing with systemic noise in fNIRS. There are two main categories of the existing approaches, which are prefilter and statistical approach (b). Those included in this work for comparison are indicated with (a) in the following section. Some of those papers used external measurements as the reference to systemic physiological responses and/or to validate their proposed method.

### 2.1 Prefiltering Methods

In this section, we will briefly detail a few of the prefiltering methods that have been previously proposed for fNIRS research and that were compared as part of this current study.

#### 2.1.1 Baseline-derived principal components analysis

PCA is a prefiltering method to reduce spatial covariance in the fNIRS data and works on the assumption that the systemic physiological signal can be identified by a strong spatial covariance structure. As introduced by Zhang et al.,^19^ the first several principal components of the spatial covariance of the fNIRS data are removed to reduce global signals. In the orignal proposed version of this, a separate baseline-only data file is used to derive the components, which are then projected from a separate data file of interest:

$$U\cdot S\cdot V^{T}=Y_{\text{baseline}}^{T}\cdot Y_{\text{baseline}},$$ (1a)

$$Y_{\text{filtered}}=Y_{\text{task}}-\sum_{i=0}^{n}Y_{\text{task}}\cdot V_{i},$$ (1b)

where U is the left singular vectors returned as the columns of a matrix; S is the singular values returned as a non-negative diagonal matrix in decreasing order; V is the right singular vectors returned as the columns of a matrix, those U, S, and V computed by singular value decomposition; $Y_{\text{baseline}}$ and $Y_{\text{task}}$ are the vectors of measurements from resting data as a baseline and task; and i is the index of number components.

The assumption in this model is that spatial structure due to the systemic noise is the same between the baseline-only and task file and therefore a filter can be designed from the baseline file and applied to the task file of interest. This is applied separately for the oxy- (${\mathrm{HbO}}_{2}$) and deoxy-hemoglobin (Hb) signals since these may have differing physiological contributions and therefore differing spatial structure. In Eq. (1b), n is the number of spatial components to be removed. Following the recommendation by Franceschini et al.,^10^ we have defined n to be the number of components needed to explain 80% of the spatial covariance by removing the first few eigenvectors of the baseline signal. That paper also speculated that removing too many components would lead to potential suppression of the estimate brain signals and introduce type-II error.

#### 2.1.2 Principal components analysis

Similar to the baseline-derived PCA (bPCA) analysis, an alternative method is to use the same data file to define the components to be removed. In this case, the decomposition [Eq. (1a)] is applied from the spatial covariance of the task dataset instead of a separate baseline-only data file. Because the principal components come from the same data file containing the task-based signal, the assumption is that the global spatial features of the systemic noise comprise a stronger component of the spatial covariance compared to the task-based signal. Equations (1a) and (1b) are used with the substitution of the $Y_{\text{task}}$ (fNIRS data from the task file) in place of the separate baseline ($Y_{\text{baseline}}$) data in Eq. (1a). Both the baseline-derived and single-file versions of the PCA filter were originally included in the HOMER software program for NIRS analysis.^31^ A limitation of this approach, however, is when the evoked brain signal of interest has a spatial extent (covariance) on the same scale as the systemic physiology, which is often true when the fNIRS probe contains only a few measurement channels and covers only the area of brain activity.

#### 2.1.3 Short-separation prefiltering via unconstrained projection

Another possibility to reduce the systemic physiological noises is by recording from additional dedicated SS measurements (see Ref. 32 for review). Since the penetration depth of fNIRS measurements varies with the light source–detector spacing, SS measurements can be used to provide a local estimate of the hemodynamic changes in the skin layer, which can be used to design filters. Brigadoi and Cooper^33^ investigated this SS measurement using Monte Carlo simulations to find the optimum distance for short channel, which was determined to be 8.4 mm for adult and 2.15 mm for infant. It has been suggested that the distance between an SS channel and the long-distance (LD) channels it is designed to filter should have a center-to-center distance of no more than 15 mm.^22^

As a prefiltering method, SS data can be projected out of the longer-separation channels (brain and skin) to better isolate the brain signals:

$$Y_{\text{filtered}}=[I-X_{\text{short}}\cdot {\left(X_{\text{short}}^{T}\cdot X_{\text{short}}\right)}^{-1}\cdot X_{\text{short}}^{T}]\cdot Y_{\mathrm{long}},$$ (2)

where $X_{\text{short}}$ is a matrix constructed from the collection of one or more SS time courses. In this model, we call this “unconstrained projection “since there is no explict use of the spatial relationships of measurements or the relative sensitivity of the short and longer measurement pairs (cf., methods based on the optical forward model as described in the next section).

#### 2.1.4 Short-separation prefiltering via image reconstruction

In comparison to the use of SS measurements as an unconstrained projection operator, an alternative approach is to attempt to introduce information about the relative sensitivity of measurements to the brain and skin by way of the optical forward model, which describes how light diffuses through the tissue. In particular, Gregg et al.^34^ introduced the idea of using a basic fNIRS image reconstruction model to project out the superficial noise signals based on the expected sensitivity of each measurement to the skin and assumptions about the lower spatial frequency of the signals in the superficial layer (see Refs. 23 and 35 for similar studies). In this work, we implemented image reconstruction-based correction using the model:

$$Y_{\text{filtered}}^{T}=[I-L\cdot S\cdot {\left(S^{T}\cdot L^{T}\cdot L\cdot S+\lambda \cdot I\right)}^{-1}\cdot S^{T}\cdot L^{T}]\cdot Y^{T},$$ (3)

where L is the partial optical forward model (matrix) describing the sensitivity of all fNIRS measurements (both short distance and LD) to a skin layer, λ is the stabilizing hyperparameter, and S is a low-spatial frequency basis set to impose spatial smoothing on the reconstructed image of the skin. For example, S could be constructed from a Gaussian smoothing kernel for the skin layer^36^ or spatial wavelets.^37^^,^^38^

### 2.2 Statistical Methods

Compared to the prefiltering methods described above, the second category of analytic methods incorporates the corrections into the statistical model in a single step. Specifically, in most fNIRS brain studies, a linear regression model of some form is used. We note that this statement covers deconvolution, canonical regression, and block averaging as the three common variations of a linear regression model used in fNIRS.^39^ For discussion of the relationship of block-averaging to the weighted back-projection solution of the equivalent regression model in the limit of nonoverlapping events (see Ref. 40). In these statistical correction models, rather than applying a separate preprocessing step to remove noise, terms are added in the regression model to remove systemic physiology. In this case, the regression statistics include estimates for the effect of these superficial terms.

#### 2.2.1 Short-separation regression models

An alternative for using SS measurements to prefilter the data is to include it as additional regressors (-of-no-interest) in the linear model. In the same manner as the prefiltering methods previously described, a combination of one or more SS channel time courses can be included into a matrix ($X_{\text{short}}$) that is concatenated to the regression matrix (design model; $X_{\text{task}}$) describing the task-based regressors:

$$Y=[\begin{array}{cc} X_{\text{task}} & X_{\text{short}} \end{array}]\cdot [\begin{array}{c} \beta_{\text{task}}\\ \beta_{\text{short}} \end{array}]+\varepsilon ,$$ (4)

where $\beta_{\text{task}}$ and $\beta_{\text{short}}$ are the activity strength of task activation and SS channel, respectively, and ε is the error terms. Of note, one limitation of this approach is when the physiology revealed by the SS measurements shows task-induced changes, this can create collinearity issues in the overall model, which destabilize estimates of the evoked signal.

#### 2.2.2 Generalized linear models

Generalized linear models, which introduce transformations of the model via prewhitening^8^^,^^26^ or precoloring,^26^ can be applied to solve the regression model and reduce false-positive rates (FPRs) introduced by serially correlated noise in the data. A detailed review of this topic is provided by Huppert.^5^ In this work, we compared OLS approach and AR-IRLS model described by Barker et al.^8^ In brief, the AR-IRLS model is first solved using robust regression and the residual noise, then fit to an AR model. The AR filter (computed by an Akaike model order) is applied to both sides of the original linear regression model and then resolved and repeated until convergence (see Ref. 5 for discussion).

#### 2.2.3 Mixed-effects models

Finally, in this work, we introduce an ME version of the linear regression model. The motivation for this model was that in the standard regression model, the weights of the SS terms are estimated independently for each LD channel of interest. Thus there is no part of the standard regression model that imposes that the SS contributions are expected to be more global and consistent across the fNIRS probe. In the ME model, we assume that these weights for the SS regression terms all come from a single-distribution pooled across all the channels. This is done in an iterative and data-driven way, which empirically determines the variance of this distribution. If the systemic response is experimentally very global, then the distribution will become narrow and the SS regression coefficients for a given channel of interest will be more statistically informed by the other channels. Conversely, if the systemic response is very heterogeneous, the variance of this prior distribution becomes very broad and this is a noninformative statistical prior. The ME regression model is given by the following equations:

$$Y=X_{\text{task}}\cdot \beta +Z_{\text{short}}\cdot \Gamma +\varepsilon ,$$ (5a)

$$\Gamma \in N(0,\sigma^{2}).$$ (5b)

Note $Z_{\text{short}}$ is the same as the previously defined $X_{\text{short}}$ but is renamed (X→Z) here to reflect the common usage of the terminology in an ME model. The random effects coefficients (Γ) are assumed to have a zero-mean normal distribution with variance $\sigma^{2}$, which is pooled across the entire probe (all channels). In this work, we use an iterative expectation–maximization approach to solve this model. (i) Given an initial guess of $\sigma^{2}$ (initially set to infinity), the variance weighted solution to Eq. (5a) is solved to estimate the task coefficients (fixed effects; β) and physiology regressors (random effects; Γ) for each LD fNIRS source–detector channel. (ii) Next, the estimate of the variance is updated by computing the mean absolute deviation (MAD) of the estimates of the random effects across the channels (σ=1.4826*MAD). The MAD is used to maintain the zero-mean estimate. (iii) The model is then re-estimated, and the processes repeated until convergence. By performing the regularized ME estimation, we are also able to stabilize the collinearity issues associated with task-induced superficial physiological changes. This also imposes a prior on the physiological noise estimates by assuming the coefficients come from a single spatially global variance distribution.

## 3 Materials and Methods

In this work, we compared the performance of the various physiological correction methods listed above. The main contribution of this paper is to compare the performance of the existing methods for removing the systemic physiological noises. To our knowledge, there has not been a full quantitative comparison of existing method to remove the systemic physiological effects in fNIRS signal using sensitivity–specificity analysis.

### 3.1 Subjects

In order to provide data for testing the performance of the various models, 12 subjects participated in the experiment (5 males, 7 females; age range 20 to 50 years; all right-handed). The subjects were informed about the experimentation and written consent was obtained. This study was approved by the University of Pittsburgh Institutional Review Board.

Each subject performed seven scans consisting of one resting and six task scans. The subjects performed the experiment with their open eyes for both resting and task sessions. The task sessions (i.e., walking, imagine walking, and BH) consisted of a 25-s task period followed by a 30-s rest period and was repeated 5 times.

The duration of the entire experiment was about 35 min. Subjects were instructed on how to complete the paradigm. First, for the RS, the subjects were to avoid body motion and to remain relaxed in the standing position for 5 min without employing any mental effort. Next, for the walking task, subjects were verbally instructed to walk at the pace of the treadmill (5 km per hour). For the imagine walking, subjects were verbally instructed to stand still but imagine walking. Finally, for the BH task, the subjects were verbally instructed to hold their breath for 25 s. At the beginning of the test, subjects were secured into a harness attached to a support to ensure their safety. The experimenter gave auditory verbal commands to begin each task. The seven tasks were done into following order: RS, walking task 1, imagine walking 1, BH 1, walking task 2, imagine walking 2, and BH 2. The walking and imagined walking tasks were not included in the main analysis of this study, but they are presented in Figs. S1 and S2 in the Supplementary Material.

The BH task triggers a vasomotor response to change in systemic oxygenation and blood flow levels. We argue this represents an extreme scenario for a physiological change. Therefore, this is the most challenging test for the SS methods. In the data processing, we used the BH data to model two different types of simulations: (i) the worst-case scenario when the physiological response is co-occurring with the task stimulus onsets (denoted BH-locked) and (ii) when physiological signals occur in the background but are random jittered with respect to the stimulus timing (denoted BH random) [see Fig. 2(b)]. In all cases, a simulated evoked response was added to half of the channels of experimental fNIRS data at a specific contrast-to-noise ratio (CNR=0.7, which was chosen as a level where the ROC analysis would not give trivial saturation effects between the models and is in line with the previous study^41^) in order to compute the ROC analysis. CNR in this work was defined as the peak magnitude of the added evoked response to the standard deviation (σ) of the oxy- or deoxy-hemoglobin data. The equation σ=1.4826*MAD [median absolute deviation] was used as a robust estimator of standard deviation to reduce the effects of strong outliers. The CNR was adjusted on a per channel basis. We also examined simulations at CNR of 0.5, 0.7, 1.0, and 2.0 for a subset of the results to examine the effect of data quality on the results. In the case of the BH-locked, those events match with the instruction to the subject when they have to hold their breath, whereas in the BH-random, we made the random events in our data processing. Thus in both cases, the level of background noise was the same. The same simulations were applied to the RS data.

### 3.2 Data Acquisition

**Fig. 1:**
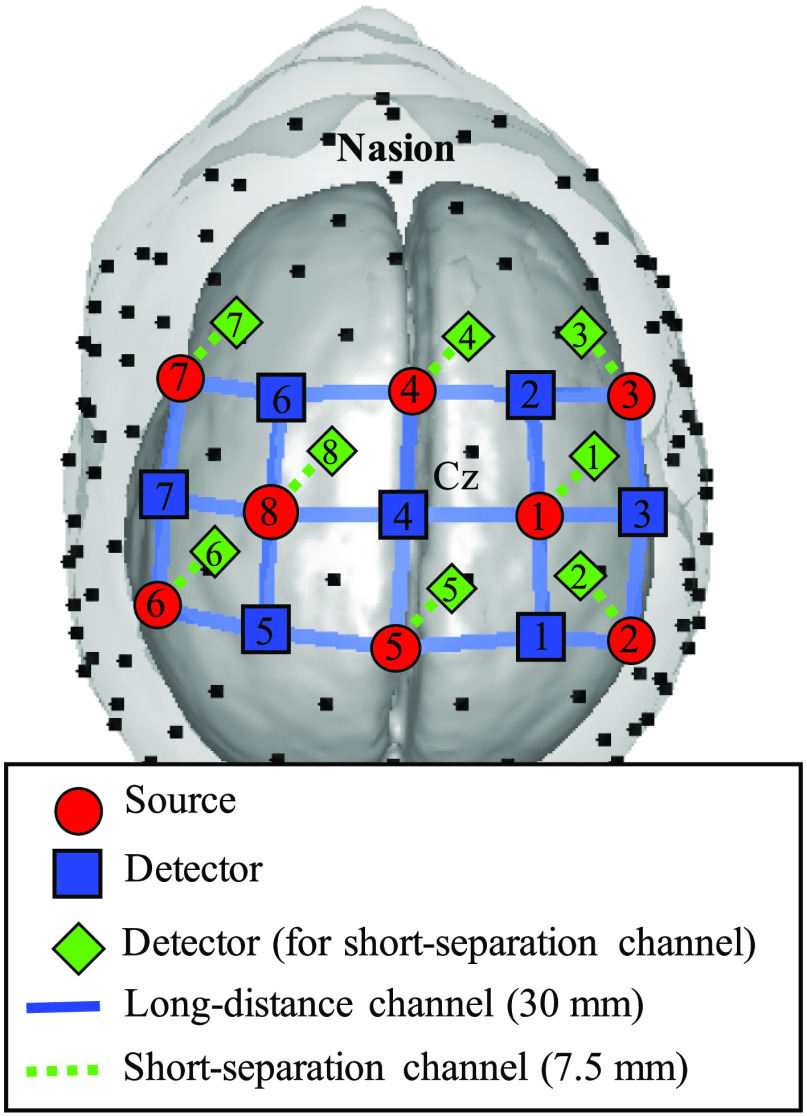
FNIRS optodes configuration. Blue solid-line and green dotted-line represent the LD (n=22) and SS (n=8) channels, respectively. Detector 4 coincides with the Cz location in the International 10–20 System.

The experimental data were recorded using a commercial NIRScout (NIRx GmbH, Berlin, Germany) continuous fNIRS system. A prototype cap using SS measurements from NIRx was used on the standard layout. The distances between source and detector were 30 and 7.5 mm for LD and SS channels, respectively. In this study, the relative center-to-center distance between SS and nearest LD channels is between 13.4 and 15.5 mm. The data were recorded at a sampling rate of 7.8125 Hz for two wavelengths (760 and 850 nm). As Fig. 1 shows, a total of 30 channels (22 channels for LD and 8 channels for SS channels) were measured from 8 sources (red circle), 7 detectors for LD channel (blue rectangle), and 1 detector (split to 8 detectors) for SS channel (green diamond). The blue solid-line represents the LD channel and the green dotted-line is the SS channel. All the optodes were placed on the scalp above the motor cortex area. Detector 4 was set to coincide with the Cz location in the international 10–20 system, as shown in Fig. 1. The room lights were turned off during the experiment to minimize signal contamination from the ambient light. Additionally, the ambient light was blocked using an opaque scuba diving cap over the cap holding the optodes.

### 3.3 fNIRS Data Processing

**Fig. 2:**
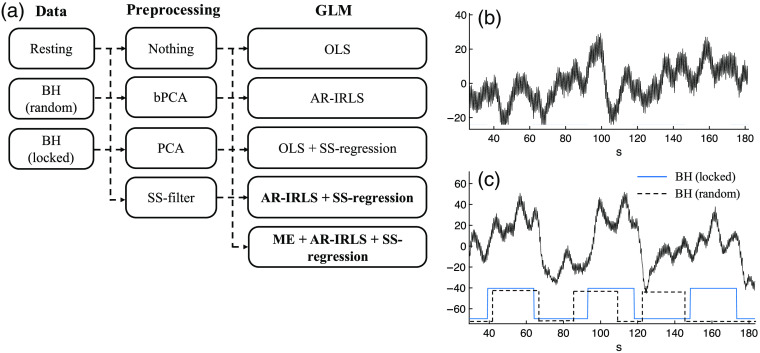
In this work, many combinations of preprocessing and statistical models (GLM) were applied to the three types of simulated data. (a) The 60 (3×4×5) combinations of processing that were used in this study. Examples of (b) the experimental resting and (c) BH data are shown, which were used to generate the three types of semisynthetic data sets by adding known evoked responses to a subset of the experimental data. (c) The timing of the stimulus events used in the BH-locked and BH-random simulations.

Several publications have described the processing technique to reduce the systemic physiological noises in Sec. 2 (see Table 1). These algorithms are mainly focused in the implementation of data processing to get better topographic and/or tomographic images. Figure 2(a) summarizes the possible or common filtering solutions with/without SS channels when reducing the systemic physiological noise. There are three major stages in the processing. (i) Three datasets contain observations from two different tasks: resting, BH (random), and BH (locked) data. This processing includes converting the raw data to hemoglobin data (${\mathrm{HbO}}_{2}$ and Hb) using the modified Beer–Lambert law. (ii) There are four different preprocessing steps in the second stage: without preprocessing, bPCA, PCA, and SS channel as a prefilter. In addition, there are two types of SS as filter: via unconstrained projection and via image reconstruction. It is noted that the SS filter via unconstrained projection as a prefiltering step is the common procedure in the literature (see Table 1). (iii) In the third stage, GLM analysis is implemented with several different algorithms: OLS and AR-IRLS with/without SS channels as a regression and ME processing using AR-IRLS with SS channel. In this study, we also compared the regression models with either oxy-, deoxy-hemoglobin $[X_{\text{short}\_({\mathrm{HbO}}_{2}|\mathrm{Hb})}$] and both [$X_{\text{short}\_({\mathrm{HbO}}_{2}+\mathrm{Hb})}$]. In this study, a total of 60 (3×4×5) combinations of processing were performed in ROC simulation [see Fig. 2(a)]. Furthermore, PCA regression was applied to remove collinearity in the $X_{\text{short}}$ matrix. For PCA regression, a orthonormal decomposision of the $X_{\text{short}}$ matrix is used and the nonzero components are used as a reparameterization of the components. This strategy is to avoid multicollinearity problem in regression analysis, which occurs when two or more independent variables are highly correlated. This was only used to remove collinearity from using multiple short-seperation channels in the model, but did not reduce any collinearity between the task regressors and the short-seperation nuisance regressors. However, since the short-seperation regressors are not masked by the on/off periods of the events in the same way that the task regressor terms are, we did not find strong mathematical collinearity between the $X_{\text{task}}$ and $X_{\text{short}}$ regression terms. In addition, the nearest n-channels (up to all SS channels) have been processed for SS investigation in the regression model. The performance of each processing combination was compared using ROC analysis in order to examine the sensitivity–specificity of the processing technique and control of type-I error.

### 3.4 Short-Separation Processing

There are two ways to process the SS channels, that is, (i) SS channels as prefiltering methods and (ii) SS channels as regression for solving GLM. For both methods, the definition of $X_{\text{short}}$ can be flexible and in this work we examined the case where $X_{\text{short}}$ was composed of either oxy-or deoxy-hemoglobin [$X_{\text{short}\_({\mathrm{HbO}}_{2}|\mathrm{Hb})}$] or both [$X_{\text{short}\_({\mathrm{HbO}}_{2}+\mathrm{Hb})}$]. We also examined both where multiple SS channels were directly used as columns of $X_{\text{short}}$ and where a PCA regression was applied to remove collinearity in the $X_{\text{short}}$ matrix (e.g., first m principal components computed from the decomposition of the nearest n SS channels where m≤n). In addition, for SS as regression, we varied the SS channels to include only the nearest n-channels up to the entire probe.

Figure 2(b) depicts the ${\mathrm{HbO}}_{2}$ for resting data. Figure 2(c) illustrates the difference between BH (locked) and BH (random) stimuli (lower figure) using ${\mathrm{HbO}}_{2}$ data for BH task (upper figure). The BH (locked) stimuli (blue solid line) means that the timing of these simulated events matched the BH timing. Meanwhile, the BH (random) stimuli (black dashed line) means that onset and interstimulus interval between the simulated 25-s duration events were jittered randomly (the interstimulus interval was jittered between 15 and 50 s) with respect to the actual BH. We generated the random stimuli in resting data in our ROC simulation (see the next section).

All these different techniques including a demo script for this study were implemented in MATLAB™ (Math-works, Natick, MA) 2018a as the part of an open-source AnalyzIR toolbox.^42^ This toolbox is currently available online^43^ or by request to the corresponding author.

### 3.5 Sensitivity–Specificity Simulation

The ROC curve is a graphical plot frequently used to evaluate the performance of the various algorithms. These curves are generated by simulating evoked responses in exactly half of the data by adding a synthetic “activity” to the experimentally recorded baseline data at a specific CNR. After running the data through the proposed analysis pipeline, the true positive and false positive channels are tallied. This is repeated for thousands of repeated random selections. In this study, for each iteration of the simulation, a resting, BH (random), and BH (locked) dataset are randomly chosen from the entire dataset collected from the 12 subjects. Then a simulated “activity” response was generated and added to exactly (randomly selected) half of the LD channels and then the same data are analyzed through the 20 (4 preprocessing×5 GLM models) algorithm pipelines. After solving the GLM model, the null hypothesis of the β for each channel is zero, which can be tested using a Student-t test. The p-values reported by this test can be used to calculate the FPR (type-I error rate) for noise-only channels and the true-positive rate (TPR) for the simulated response-containing channels given a p-value threshold (p-hat). In the AnalyzIR toolbox,^42^ the ROC analysis is performed for both ${\mathrm{HbO}}_{2}$, Hb, and joint test (Hotelling’s $T^{2}$ test) of ${\mathrm{HbO}}_{2}\text{–}\mathrm{Hb}$. However, in this study, we often show the HbO_2_ result since the AUC of ${\mathrm{HbO}}_{2}$ slightly higher than Hb. All true positives are generated by adding simulated brain stimuli to resting or BH data. In each iteration of simulation under resting or BH condition, the data of a randomly selected subject under this specific condition are used as the physiological signal for simulation. ROC analysis allows a complete sensitivity and specificity report in a coordinate system.

An ROC curve is a plot of TPR (also known as sensitivity) versus FPR (or 1-specificity) at various threshold settings. Each point on the ROC curve expresses the sensitivity–specificity pair corresponding to a particular decision threshold. Here, we used 1−p-value as the decision threshold since a smaller p-value indicates the β is more significantly different from zero. The accuracy is measured by the AUC whose statistical meaning is the probability the algorithm ranks a randomly chosen positive (stimulus-containing) case higher than a negative (noise-only) case in 1−p-value, i.e., lower in p-value. Thus the AUC value of 1 represents a perfect test and AUC of 0.5 represents random chance. Furthermore, we also estimated the control of the type-I error by showing the relationship between empirical p-value (actual FPR extracted from the ROC curve) against the theoretical FPR (denoted p-hat). If the null hypothesis is true, an appropriate analysis approach or statistical test should provide p-values uniformly distributed from 0 to 1. In this case, the relationship is displayed as points on an x axis (i.e., p-hat) and y axis (i.e., actual FPR) coordinate system. The ideal condition (“truth”) shows an identical value between p-hat and FPR value, where the slope of that condition is equal to 1. A large positive deviation means the model over-estimates the significance of events. Meanwhile, a dip below the slope of unity means the model underestimates the significance of the results.

### 3.6 Effect of Short-Separation Data Quality

In addition to looking at the model performance using multiple SS channels, we also examined the effect of the data quality of the SS channel. To do this, we added each single SS (1 of 8) channel to the regression model and ran simulation ROC analysis for each data file. The quality of the SS data was quantified using the scalp-coupling index (SCI) approach described in Ref. 44, which is based on cross correlation of the two wavelengths of fNIRS data around the cardiac frequency. The AUC for the ROC analysis was examined for datasets using SS data with SCI in 0.10 bins from 0 to 1. For these simulations, only the BH data were used with a randomly jittered simulated block design activation at a CNR of 0.7. Only the AR-IRLS version of the GLM was examined.

## 4 Results and Discussion

For this study, we investigated 60 different processing combinations using three datasets as shown in Fig. 2. Additionally, we also examined the SS channels as regressors and as a prefilter model to include only the nearest n channels up to the entire probe. These additional models are presented in the Supplementary Material for SS as a prefilter. In this main text, only the results of using the entire set of 16 SS channels as a prefilter (8 channels by ${\mathrm{HbO}}_{2}/\mathrm{Hb}$) are presented. All of the processes were simulated using our NIRS AnalyzIR toolbox.^42^ The ROC analysis (sensitivity–specificity report) and control type-I error (p-hat and FPR report) was run from 1650 iterations of each processing pipeline.

### 4.1 ROC and Control Type-I Error Curves

**Fig. 3:**
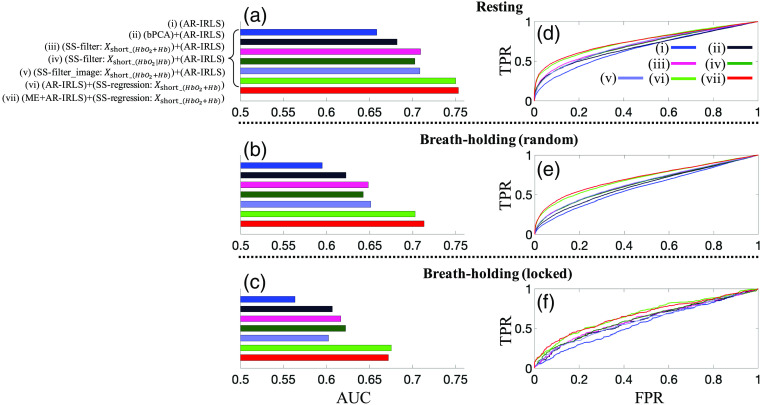
Comparison of (a)–(c) the AUC from (d)–(f) sensitivity–specificity ROC curve. In every panel, six selected different processing pipelines are investigated. (a), (d) Resting data; (b), (e) BH with random stimulus marking; and (c), (f) BH with locked stimulus marking.

Figures 3(a)–3(c) depict the AUC values from the ROC curves (sensitivity–specificity reports), in the right panels (d)–(f), of the selected seven different processing pipelines using AR-IRLS. From the 60 possible combinations (shown in Fig. 2), we selected 7 different processing pipelines for presentation: (i) AR-IRLS without preprocessing (blue), (ii) preprocessing using bPCA and AR-IRLS (black), (iii) prefilter using SS channels (SS-filter) [$X_{\text{short}\_({\mathrm{HbO}}_{2}+\mathrm{Hb})}$] via unconstrained projection as a filter and AR-IRLS (magenta), (iv) prefilter using SS channels (SS-filter) [$X_{\text{short}\_({\mathrm{HbO}}_{2}|\mathrm{Hb})}$] via unconstrained projection as a filter and AR-IRLS (dark-green), (v) preprocessing using all SS channels $[X_{\text{short}\_({\mathrm{HbO}}_{2}+\mathrm{Hb})}$] via image reconstruction as a filter and AR-IRLS (light-blue), (vi) AR-IRLS with SS channels $X_{\text{short}\_({\mathrm{HbO}}_{2}+\mathrm{Hb})}$] as a regression (SS-regression) (green), and (vii) ME and AR-IRLS with SS-regression $X_{\text{short}\_({\mathrm{HbO}}_{2}+\mathrm{Hb})}$] (red). It is noted that the SS-filter and SS-regression are using all SS channels for this figure. In this figure, we only showed and compared those various processing pipelines using AR-IRLS since we know from the previous works that the AR-IRLS has higher AUC than OLS.^8^^,^^42^ The resting, BH with randomly jittered event timing (BH-random), and BH with events time-locked to the hold (BH-locked) data are shown in the first [panels (a) and (d)], second [panels (b) and (e)], and third row [(panels (c) and (f)], respectively.

Figure 3 compares the performance of the processing with/without SS channels. We found that, in general, the processing performed better using additional SS channels as a prefilter (SS-filter) in the prefilter as well as a regression (SS-regression) for solving GLM. We observed that the approaches that used SS data either as a prefiltering step or within the linear model were better than the PCA or bPCA methods or regression without SS. Thus SS data should be recorded when possible. If SS data are not available, the bPCA method was the best alternative. When using SS data, we found that regression approaches that incorporate these into regressors-of-no-interest in the statistical model were better than using these as part of a prefiltering step. We also noted that using both hemoglobin species in the regression model was better than using only the species corresponding to the LD data of interest. Finally, we found that our new ME variation of the GLM did have a slight improvement but since this algorithm is iterative and was found to take around 10-fold longer to solve, the modest improvement of this approach was felt to be impractical.

As expected, the performance of all the methods was best for the simulations involving just the RS data and the AUC estimates were lower for the BH data with randomly jittered event timing and lowest for the BH data with time-locked events. The values for all comparisons are provided in Table 2. By performing a statistical test on the AUC difference using the method proposed by DeLong et al.,^45^ we found the AUCs of the two methods using SSs as regressors [see Fig. 3, (vi) and (vii)] are significantly larger than those of the remaining methods with p-values<0.05. Since, in general, one mostly cares about the range of p<0.05 in the context of reporting scientific findings, we further examined the partial AUC of the models for the range of FPRs between 0 and 0.05. In this range, as shown in Fig. 3, the models show the largest differences. The partial area under the ROC curve (pAUC)^46^ with FPR≤0.05 was used as the performance index for each method. For the RS data, the normalizes of pAUC for SS-regression + AR-IRLS [see Fig. 3(vi)] and SS-regression + ME + AR-IRLS [see Fig. 3(vii)] are 0.32 [raw pAUC=0.016] and 0.34 [raw pAUC=0.017], respectively; whereas the pAUC for SS-filter image [see Fig. 3(v)] is 0.20 [raw-pAUC=0.010]. By estimating the pAUC variance using bootstrap,^46^^,^^47^ the Student’s t-test for the pAUC difference between the SS-regression and SS-filter method was conducted, which suggested significant difference in pAUC with p-values of $p<10^{-6}$ for the SS-regression + AR-IRLS method and $p<10^{-6}$ for the SS-regression + ME + AR-IRLS method both in comparison to the SS-filter method.

**Table 2:** AUC values for various processing pipelines using AR-IRLS

Data	Preprocessing	AR-IRLS	SS-filter^a^ + AR-IRLS	SS-filter^b^ + AR-IRLS	SS-filter image^a^ + AR-IRLS	SS-regression^a^ + AR-IRLS	SS-regression^a^ + ME + AR-IRLS
Resting	None	0.66	0.71	0.70	0.71	0.75	0.75
	PCA	0.68	0.71	0.72	0.69	0.76	0.76
	bPCA	0.68	0.72	0.71	0.70	0.75	0.75
BH (random)	None	0.60	0.65	0.64	0.65	0.70	0.71
	PCA	0.63	0.67	0.67	0.66	0.72	0.72
	bPCA	0.62	0.66	0.66	0.63	0.70	0.71
BH (locked)	None	0.56	0.62	0.62	0.60	0.68	0.67
	PCA	0.57	0.63	0.63	0.63	0.67	0.67
	bPCA	0.61	0.65	0.64	0.62	0.68	0.68

a Both of oxy- and deoxy-hemoglobin [$X_{\text{short}\_({\mathrm{HbO}}_{2}+\mathrm{Hb})}$].

b Either of oxy- or deoxy-hemoglobin [$X_{\text{short}\_({\mathrm{HbO}}_{2}+\mathrm{Hb})}$].

**Fig. 4:**
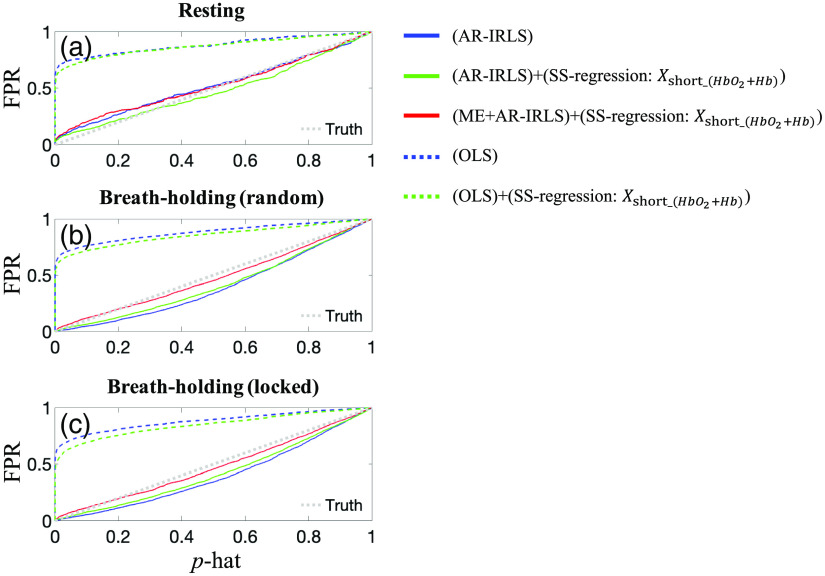
Comparison of OLS and AR-IRLS of control type-I error reports from three different data sets using selected six different processing pipelines. (a) Resting data, (b) BH with random stimulus marking, and (c) BH with locked stimulus marking.

In analysis shown in Fig. 3 and Table 2, we found similar results between the AR-IRLS (autoregressively iterative robust least squares) model and OLS (not-shown) regression in terms of the AUC. In general, there was only a modest improvement for the AR-IRLS approach in the AUC, which is consistent with our previous work for fNIRS data with little to no motion-artifacts.^8^^,^^42^ As previously detailed, the AR-IRLS model robust statistical estimator and differences in the AUC of these methods is only expected in the presence of statistical outliers (e.g., motion artifacts). However, as shown in Fig. 4, these two methods have substantially different sensitivities to serially correlated noise, which causes high FPRs and uncontrolled type-I error. Figure 4 shows the calculated FPRs against the p-hat at various thresholds from the ROC curves. We compared the performance of the control type-I error between OLS (dashed-line) and AR-IRLS (solid-line) for three different processing pipelines (i.e., without preprocessing, SS-regression, and SS-regression followed by ME for AR-IRLS only). Those figures also compare three resting, BH (random), and BH (locked) datasets as shown in panels (a)–(c), respectively. Overall, AR-IRLS had a substantial significant improvement on the control for type-I error rates for all three datasets. OLS processing with/without SS channels had high FPR or type-I error (see dashed-line). This result shows that the use of SS channels as regression for OLS will not substantially improve control of type-I errors. For example, in resting data, the FPR at the p-hat<5% are 70% and 64% for OLS and OLS with SS-regression, respectively. This means that at an expected threshold of p<0.05, the actual FPRs are around 60% to 70%. Meanwhile, AR-IRLS performed better than OLS in control of type-I error (see solid line). The processing using AR-IRLS is very close to the ideal case (where the reported p-value value is the same as the FPR). For the AR-IRLS model, the FPR at the p-hat<5% are 5% and 4% for AR-IRLS and AR-IRLS with SS-regression, respectively. This finding is in agreement with the findings from Barkeret al.,^8^ which showed that the AR-IRLS performed better than OLS in the presence of physiological noise. AR-IRLS improves control of type-I errors, which shows the FPR was reduced to 40% to 60% compared with OLS. For ME model, either OLS or AR-IRLS with SS-regression has similar control type-I error (see green dashed-line and dark green solid-line).

For the RS, the control of type-I errors in the AR-IRLS model was near prefect. However, for the BH data (both random and locked), the FPR of the AR-IRLS was elevated (e.g., 10% to 15% at p<0.05), but this was still substantially lower than the 70% FPR (at p<0.05) of the OLS method for this same data.

### 4.2 ROC Performance for Different CNR Levels

**Fig. 5:**
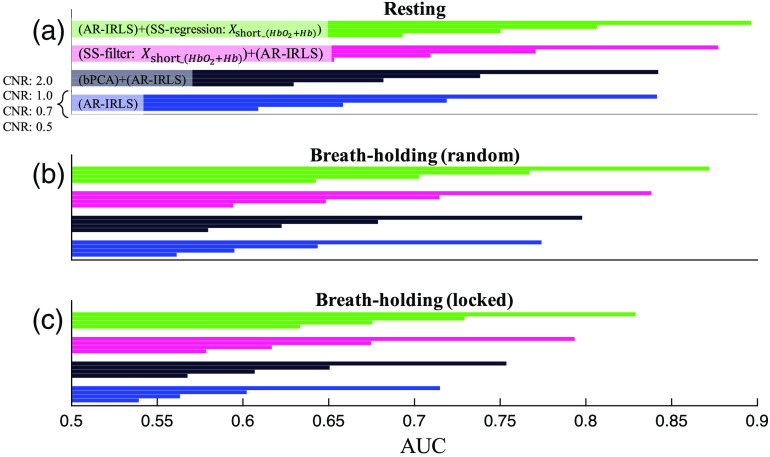
Comparison of AUC from ROC analysis at various CNRs of 0.5, 0.7, 1.0, and 2.0 from selected four different processing pipelines. (a) Resting data, (b) BH with random stimulus marking, and (c) BH with locked stimulus marking.

The results in Figs. 3 and 4 showed simulations at a CNR of 0.7. In Fig. 5, we also investigated the performance of ROC analysis with various CNR levels from 0.5 to 2.0. Four different processing pipelines have been compared in every panel: AR-IRLS without preprocessing (blue), preprocessing using bPCA and AR-IRLS (black), prefilter using SS channels (SS-filter) [$X_{\text{short}\_({\mathrm{HbO}}_{2}+\mathrm{Hb})}$] via unconstrained projection as a filter and AR-IRLS (magenta), and AR-IRLS with SS channels [$X_{\text{short}\_({\mathrm{HbO}}_{2}+\mathrm{Hb})}$] as a regression (SS-regression) (green). In all pipelines, the AUC values increased at higher CNR levels. It is expected that as CNR increases even more, the AUC of the ROC plots will begin to saturate near AUC=1. However, over the physiological range of CNR between 0.5 and 2.0, the AR-IRLS method with SS channels was the best approach.

### 4.3 Number of SS Channels

An open question in the previous work has been whether a separate SS channel is necessary for every LD channel, or whether a single or limited number of SS channels may be sufficient to measure the systemic physiological noises. If the systemic physiology is global, then having many SS channels would likely be redundant. However, if the heterogeneity in blood vessel sizes, volume fraction, or geometry may be different across the head, in which more SS channels would be beneficial as suggested in the previous work by Ref. 22. To address this, we ran the ROC analysis and compared the performance of the various processing pipelines using the nearest SS channels from one- or two- up to eight-channel.

**Fig. 6:**
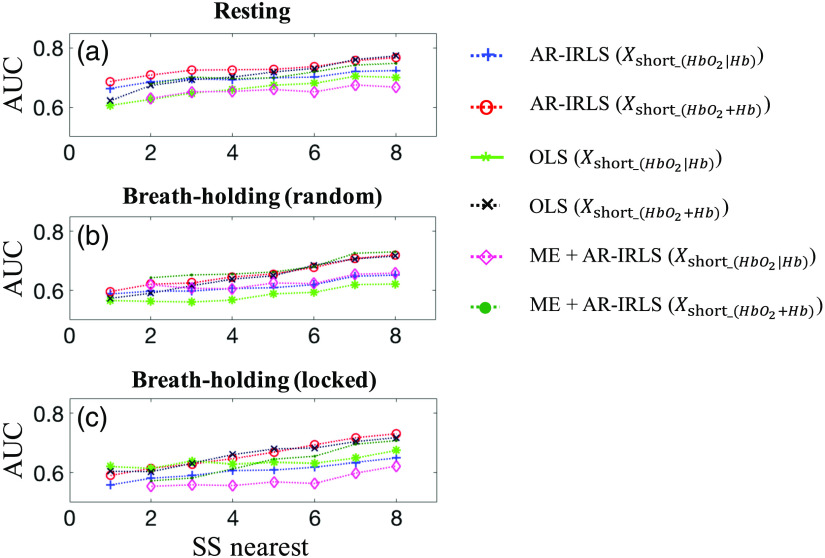
Comparison of sensitivity–specificity of AUC without preprocessing using various nearest SS channels for six selected processing. All these processing have been implemented using three datasets: (a) resting, (b) BH (random), and (c) BH (locked) data.

Figure 6 displays the AUC values of the sensitivity–specificity reports from various SS channels as regression for solving GLM (one or two nearest up to eight SS channels) without preprocessing. Those figures also compare three different datasets: resting, BH (random), and BH (locked) data as shown in panels (a)–(c), respectively. In this figure, we selected six different processing pipelines: AR-IRLS [$X_{\text{short}\_({\mathrm{HbO}}_{2}|\mathrm{Hb})}$] (blue plus), AR-IRLS [$X_{\text{short}\_({\mathrm{HbO}}_{2}+\mathrm{Hb})}$] (red circle), OLS $X_{\text{short}\_({\mathrm{HbO}}_{2}|\mathrm{Hb})}$] (green asterisk), OLS [$X_{\text{short}\_({\mathrm{HbO}}_{2}+\mathrm{Hb})}$] (black cross), ME + AR-IRLS [$X_{\text{short}\_({\mathrm{HbO}}_{2}|\mathrm{Hb})}$] (magenta diamond), and ME + AR-IRLS [$X_{\text{short}\_({\mathrm{HbO}}_{2}+\mathrm{Hb})}$] (dark green point). It is noted that the ME model needs at least two SS channels when it is solving the GLM. We only showed the use of SS channels as regression to investigate the nearest SS channels since we know from the previous results that the SS-regressing has better performance than SS-filter (see Fig. 3 and Table 2). For all simulations, data not shown, we found that OLS has higher FPRs (type-II error) similar with the previous figure (see Fig. 4).

Overall, as shown in Fig. 6, the AUC values are increased accordingly as the processing has more SS channels. Meanwhile, by increasing the number of SS channels, the performance has slightly increased in AUC, which is indicated by a decrease of the FPR (data not shown). The top two AUC values among six different processings without preprocessing are AR-IRLS [SS-all: $X_{\text{short}\_({\mathrm{HbO}}_{2}+\mathrm{Hb})}$] (0.77, 0.72, and 0.73) and ME [SS-all: $X_{\text{short}\_({\mathrm{HbO}}_{2}+\mathrm{Hb})}$] (0.75, 0.73, and 0.71) for resting, BH (random), and BH (locked) data, respectively. Chi-squared tests^48^ show they are not significantly different with p-values 0.314, 0.617, and 0.246. However, the ME has better control of type-I error. That is, AR-IRLS [SS-all: $X_{\text{short}\_({\mathrm{HbO}}_{2}+\mathrm{Hb})}$] are 18.6% (resting), 9.1% [BH (random)], and 7.4% [BH (locked)]; and ME [SS-all: $X_{\text{short}\_({\mathrm{HbO}}_{2}+\mathrm{Hb})}$] are 16.5% (resting), 6.4% [BH (random)], and 5.4% [BH (locked)] at a threshold of p<0.05. It is also suggested that it is better to use $X_{\text{short}\_({\mathrm{HbO}}_{2}+\mathrm{Hb})}$ for various nearest SS channels, which is similar with the previous finding in Fig. 3. However, the control of type-I error has slightly better performance using AR-IRLS [$X_{\text{short}\_({\mathrm{HbO}}_{2}+\mathrm{Hb})}$]; that is, 19.9% (resting), 10.6% [BH (random)], and 8.2% [BH (locked)] using AR-IRLS [$X_{\text{short}\_({\mathrm{HbO}}_{2}+\mathrm{Hb})}$] and 7.4% (resting), 9.1% [BH (random)], and 18.6% [BH (locked)] using AR-IRLS [$X_{\text{short}\_({\mathrm{HbO}}_{2}|\mathrm{Hb})}$] using all SS channels. The p-values of the chi-squared test^48^ for the differences between them are all <0.0001, which indicates AR-IRLS [$X_{\text{short}\_({\mathrm{HbO}}_{2}+\mathrm{Hb})}$] performs significantly better than AR-IRLS [$X_{\text{short}\_({\mathrm{HbO}}_{2}|\mathrm{Hb})}$].

In addition, we also processed all eight SS channels for all hemoglobin data (${\mathrm{HbO}}_{2}$ and Hb) by applying the singular value decomposition. We found that five SS channels are sufficient for most files (across subjects and datasets), as five channels can explain more than 90% of the variance (see the Supplementary Material). This finding supports the previous investigation for various nearest SS channels (Fig. 6), in which five SS channels substantially improved the performance. For example, in resting data {see Fig. 6(a); AR-IRLS [$X_{\text{short}\_({\mathrm{HbO}}_{2}+\mathrm{Hb})}$]}, the AUC values are 0.66, 0.69, 0.73, and 0.77 for AR-IRLS without SS channels, with one SS channel, five SS channels, and all eight SS channels, respectively. Meanwhile, the FPR of AR-IRLS (resting data) at a threshold of p<0.05 are 8.2% (without SS channel), 7.0% (one SS channel), 8.3% (five SS channels), and 8.2% (eight SS channels). In addition, the TPRs of AR-IRLS (resting data) at a threshold of p<0.05 are 27.7% (without SS channel), 32.5% (one SS channel), 43.7% (five SS channels), and 52.1% (eight SS channels). This finding further supports the idea of the optimum number of SS channels, which is sufficient to reduce the effect of systemic physiological noises. It is better to have the additional SS channels in every source position. However, we still can use several SS channels in some source position. These findings will help other researchers who have limited number of optodes to find the optimum number of SS channels for specific study.

### 4.4 ROC Performance per Quality of SS Data

**Fig. 7:**
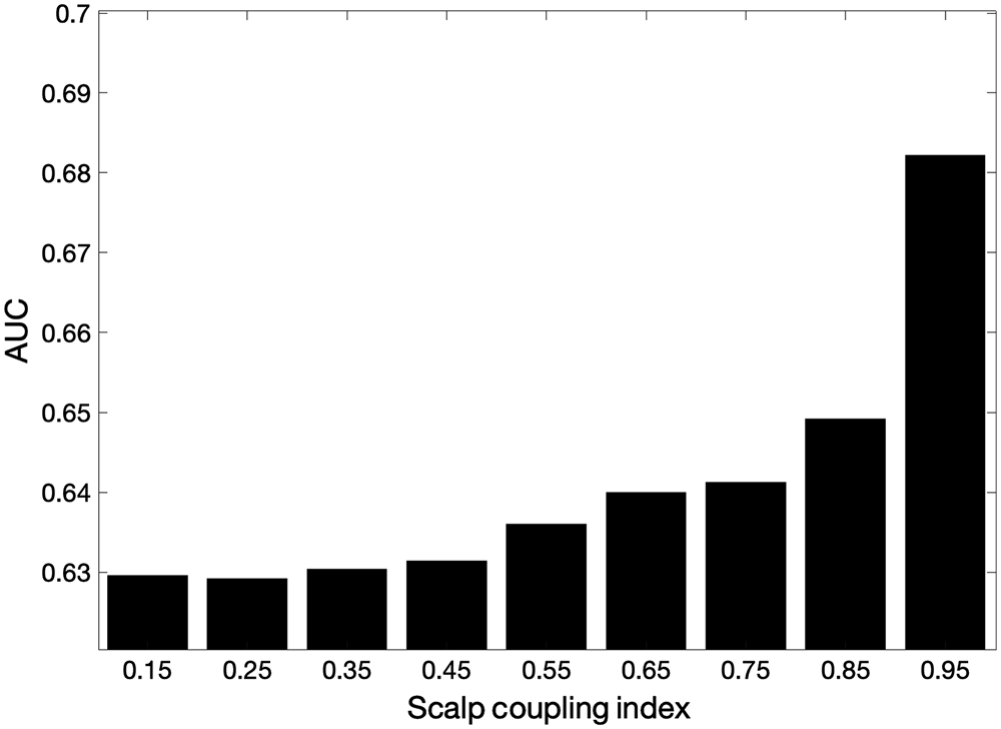
Comparison of ROC performances (AUC values) with respect to SCI values. The AUC for the AR-IRLS + SS model using SS data of varied quality. Each bar shows the AUC for data using SS channels with a specific SCI (bins of ±0.05).

We also examined the relationship of the proposed algorithms to the quality of the SS data. To address these issues, we adopted the scalp coupling index (SCI) metric by Pollonini et al.,^44^ which is based on the cross correlation of the two wavelengths of optical data for each channel around the cardiac frequency. In the best data, the cardiac signals at two wavelengths are correlated, thus yielding an SCI close to 1. In contrast, noisy cardiac signals led to a lower SCI value (close to 0). In addition, PHOEBE software used an SCI threshold of 0.8 to identify channels with acceptable scalp coupling. Bandpass filtering between 0.5 and 2.5 Hz is used to limit the signals to around the cardiac oscillation prior to compute the SCI is required. In our study, 57% of SS data had SCI more than 0.8. In order to investigate the effect of data quality, an ROC curve was computed for each of the BH data sets using only one of the eight SS channels for that data as a regressor and iterating over all SS channels. The true and FPRs were aggregated for simulations using SS channels with an SCI in bins around 0.15±0.05 to 0.95±0.05 was computed. Figure 7 shows the comparison of AUC values of the sensitivity–specificity reports from various SCI values of SS channels as regression for solving GLM using AR-IRLS.

### 4.5 ROC Analysis Using Bandpass Filtered Data

The use of SS measurements as regressors within the GLM is not exclusive of additional preprocessing. One of the common preprocessing steps in fNIRS is the use of bandpass filtering (BPF). Generally, filtering can be used to reduce the effect of background physiological noise (e.g., blood pressure fluctuations: ∼0.1    Hz, respiratory: ∼0.2 to 0.3 Hz, and cardiac: ∼1  Hz). Low-pass filtering down to about 0.5 Hz can be used routinely to remove high-frequency cardiac noise. However, the stopband on a high-pass filter needs to account for the timing of the brain activity/task as well to avoid over-filtering into the response of interest. The incorrect selection of cutoff frequency may reduce the performance of sensitivity–specificity analysis. We have previously detailed how autoregressive models embedded in the GLM offer generalized improved performance compared to high-pass filtering.^42^ In this final section, we examined the addition of both low- and high-pass filtering in addition to the use of SS regressors. In all cases, the filtering was applied to both the data and SS regressor followed by the GLM and ROC analysis. We used the BH dataset with randomly jittered additive “activity” for the simulations (BH-random)

**Fig. 8:**
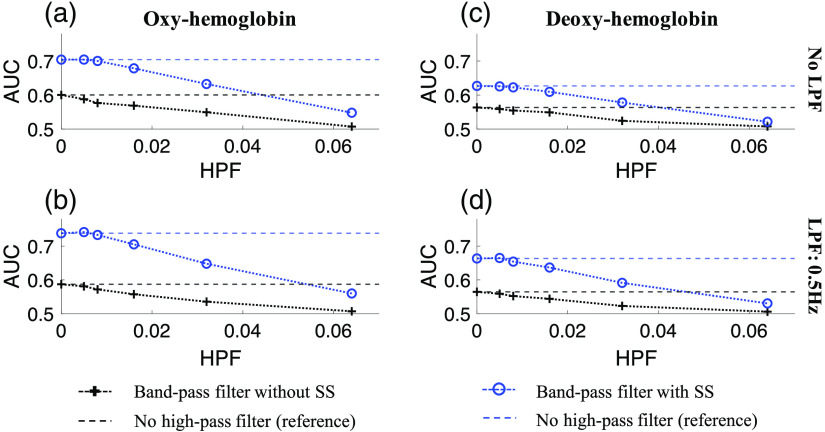
Comparison of AUC from ROC analysis at various cutoff frequencies of LPF ([0, 0.5] Hz) and HPF ([0, 0.005, 0.008, 0.016, 0.032, 0.064] Hz) from (a), (b) ${\mathrm{HbO}}_{2}$ and (c), (d) Hb. In every panel, BH (random) data using AR-IRLS with/without SS-regressor at various levels of HPF are investigated.

Figure 8 shows the comparison of the ROC analysis in BH (random data) using AR-IRLS with/without SS-regressor for both ${\mathrm{HbO}}_{2}$ [panels (a) and (b)] and Hb [panels (c), (d)]. We investigated the combination of various levels: (i) no LPF [panels (a), (c)] and LPF: 0.5 Hz [panels (b), (d)]; and (ii) HPF ([0, 0.005, 0.008, 0.016, 0.032, 0.064] Hz) [panels (a)–(d)]. Each panel shows the AUC results of the model using BPF alone (black line) and BPF with SS regression terms in the GLM (blue lines). The results of the model with no high-pass filter are shown as a dotted line across the plots for reference. As shown in this figure, we found that high-pass filtering (either with or without the use of SS regressors in the model) actually had a lower performance compared to the GLM alone. In agreement with our previous reports,^42^ the autoregressive terms in the GLM outperform a separate high-pass filter step. At low frequencies (0.005 and 0.08 Hz) for the passband of the filter, the HPF had no effect, but significantly lowered the AUC for higher frequencies where the filter is now cutting into the response of interest. Adding the low-pass filter (top row versus bottom row) did improve the AUC of the models using SS-regression compared to not filtering. However, this effect was mitigated when additional high-pass filtering was also applied. Thus we strongly recommend using the autoregressive model over a prefiltering high-pass step. In particular, using proper statistical models that are more robust to the effects of physiology and motion-artifacts (e.g., AR-IRLS), we can get reasonable AUC and we also can control FPRs (as discussed earlier) even without removing the noises using filter ahead of time.

## 5 Conclusions

In this paper, we examined the performance of various approaches to deal with the systemic physiology, which contaminates fNIRS measurements. We examined both prefiltering methods, as well as, statistical analysis methods incorporating physiological noise models into the GLM including the use of SS channels as regressors of no interest. Sensitivity–specificity analysis was used to compare the performance of the various analytic methods using synthetic additive responses (truth) added to experimental RS and BH NIRS data. These simulations were used to estimate the true positive and FPRs for the estimation of brain activity for varied algorithms, types of physiological noise, and CNRs of the activation signal. Overall, we found that the use of all available SS channel data as regressors of no interest in the statistical regression model was the best approach based on the AUC of ROC analysis. Although all three of the approaches tested to solve this regression model gave similar sensitivity–specificity results, we found that the generalized linear model using an autoregressive robust least squares (AR-IRLS) approach had far superior control of type I error in comparison to OLS methods. The third novel method of an ME variation of the AR-IRLS model, which was introduced in this work and grouped the parameter variance of the SS regressors separately from the task-based regressors, showed a slight improvement in performance, but was felt to be unjustified at this time due to a substantially higher computation cost of the iterative algorithm.

We found that the AR-IRLS regression model with SS regressors had near ideal control of type-I error for the RS data. In comparison, the OLS model suffered from extremely high uncontrolled type-I error. For example, the actual FPR at the expected threshold of p<0.05 was actually 60% to 70% for OLS. At the same threshold, the FPR was near ideal at 5% for the AR-IRLS model. When the timing of the simulated tasks was time-locked to the BH noise, this FPR for AR-IRLS jumped to 10% to 15% at p<0.05 (moderately uncontrolled type-I errors) but still well below the 70% rate of the OLS method.

In examining the question of the ideal number and quality of the SS channels, we found that the model was still improving with all eight available short channels added to the regression model and using both oxy- and deoxy-hemoglobin signals. Since our system had a maximum of eight SS channels, we do not know how much benefit could be gained by added even more such channels, but from our data were clear that using more channels improved the performance of the model in ROC analysis despite the added degrees-of-freedom associated with additional regressors. Not unexpectedly, we also found that better quality SS data (based on the SCI as a metric of quality) improved the method more than poor data. Poor data, however, did not seem to hurt the AR-IRLS regression model too much and was similar to not using any SS regressors. It is unclear if this is also true for the OLS method or the use of SSs in prefiltering steps, as the effect of data quality was not examined for these alternative methods.

Finally, we found that additional low-pass filtering could be used in conjunction with the SS regression methods to slightly improve the AUC of the ROC models for AR-IRLS regression. However, we found that high-pass filtering should be avoided. Since the autoregressive part of the AR-IRLS method already corrects for serial correlations in the data, a low level of high-pass filtering had no effect and was redundant from the effects of the AR model. However, when more aggressive high-pass filtering was used where the stop band began to cut into the response of interest, this filtering produced worse results than the AR-IRLS method alone. Thus using the AR-IRLS method, SS measurements and data should be used as-is or with low-pass filtering only applied to both the data and the regressors in the model.

### 5.1 Future Directions and Limitations

In this paper, we described a first-level statistical model using an ME variation of our AR-IRLS approach. This approach did offer some improvement over the regular AR-IRLS model, but this improvement was only slight, and we felt did not justify the additional 10-fold computation times for the iterative model. Nonetheless, this model did show the best control of type-I errors in the most difficult case of evoked responses that were time-locked to the BH events. The time-locked BH dataset (strongly coupled physiology and task) still poses significant challenges to fNIRS. The AR-IRLS and ME AR-IRLS had the best control of type-I errors in this case, but the FPRs were still higher than expected (10% to 15% at p<0.05). In comparison, the FPR was 70% at p<0.05 for OLS regression even using SS data as regressors. In some ways, this is in inherent problem of the statistical test being performed, which is testing the null hypothesis that the signal during task period is not different from zero. In this sense, the change in the signal due to evoked physiology (BH) during the task period is a valid rejection of this hypothesis and thus, is not a “false” positive for the test actually being performed. The statistics gave the right answer; we just asked the wrong question. Thus, as future work, we need to examine more specific ways to frame this hypothesis in a way that predicates the changes as originating in the brain. Still with the time-locked BH simulations as perhaps the worst-case scenario, the improvement of the FPR from 70% at p<0.05 with OLS and SS regressors to 10% with the ME AR-IRLS SS model described in this work is a step in the right direction.

This study is not without limitations. One limitation is the use of BH and RS data for background noise. These are the two extremes, but in reality, most studies are probably somewhere in between in terms of levels of physiological noise. In addition, the data we used had very little motion related artifacts, which could pose additional problems for the analysis. In the previous work, the development of the AR-IRLS as a robust* statistical estimator was shown to work well for statistical outliers due to motion artifacts; however, we do not know the effect of having motion-artifacts and outliers in the SS regressor terms. (* Robust in this context refers to the statistical definition of methods to reduce the influence of outliers.) A possible extension to this approach to deal with motion artifacts in both the data of interest and SS regressors could use the robust bivariate regression methods such as those that we have previously detailed in the context of robust correlation estimation methods for fNIRS.^49^ This, however, would need to be explored in the future work.

Finally, a limitation of this work is that all of the ROC analysis shown in this work is based on numeric simulations using experimental baseline/physiological noise but added synthetic activation “truth.” Further future work exploring these models in experimental data is still needed.

## References

[1] Jobsis F. F., “Non-invasive, infra-red monitoring of cerebral $O_{2}$ sufficiency, bloodvolume, ${\mathrm{HbO}}_{2}\text{-}\mathrm{Hb}$ shifts and bloodflow,” Acta Neurol. Scand. Suppl. 64, 452–453 (1977). ANSLAC 0065-1427 268870

[2] Boas D. A. Dale A. M. Franceschini M. A., “Diffuse optical imaging of brain activation: approaches to optimizing image sensitivity, resolution, and accuracy,” Neuroimage 23, S275–S288 (2004). NEIMEF 1053-8119 10.1016/j.neuroimage.2004.07.011 15501097

[3] Ferrari M. Quaresima V., “A brief review on the history of human functional near-infrared spectroscopy (fNIRS) development and fields of application,” Neuroimage 63(2), 921–935 (2012). NEIMEF 1053-8119 10.1016/j.neuroimage.2012.03.049 22510258

[4] Yucel M. A. et al., “Functional near infrared spectroscopy: enabling routine functional brain imaging,” Curr. Opin. Biomed. Eng. 4, 78–86 (2017). 10.1016/j.cobme.2017.09.011 29457144 PMC5810962

[5] Huppert T. J., “Commentary on the statistical properties of noise and its implication on general linear models in functional near-infrared spectroscopy,” Neurophotonics 3(1), 010401 (2016). 10.1117/1.NPh.3.1.010401 26989756 PMC4773699

[6] Tachtsidis I. Scholkmann F., “False positives and false negatives in functional near-infrared spectroscopy: issues, challenges, and the way forward,” Neurophotonics 3(3), 031405 (2016). 10.1117/1.NPh.3.3.031405 27054143 PMC4791590

[7] Zhang Q. Strangman G. E. Ganis G., “Adaptive filtering to reduce global interference in non-invasive NIRS measures of brain activation: how well and when does it work?” Neuroimage 45(3), 788–794 (2009). NEIMEF 1053-8119 10.1016/j.neuroimage.2008.12.048 19166945 PMC2671198

[8] Barker J. W. Aarabi A. Huppert T. J., “Autoregressive model based algorithm for correcting motion and serially correlated errors in fNIRS,” Biomed. Opt. Express 4(8), 1366–1379 (2013). BOEICL 2156-7085 10.1364/BOE.4.001366 24009999 PMC3756568

[9] Barker J. W. et al., “Correction of motion artifacts and serial correlations for real-time functional near-infrared spectroscopy,” Neurophotonics 3(3), 031410 (2016). 10.1117/1.NPh.3.3.031410 27226974 PMC4876834

[10] Franceschini M. A. et al., “Diffuse optical imaging of the whole head,” J. Biomed. Opt. 11(5), 054007 (2006). JBOPFO 1083-3668 10.1117/1.2363365 17092156 PMC2637816

[11] Santosa H. et al., “Noise reduction in functional near-infrared spectroscopy signals by independent component analysis,” Rev. Sci. Instrum. 84(7), 073106 (2013). RSINAK 0034-6748 10.1063/1.4812785 23902043

[12] Mesquita R. C. Franceschini M. A. Boas D. A., “Resting state functional connectivity of the whole head with near-infrared spectroscopy,” Biomed. Opt. Express 1(1), 324–336 (2010). BOEICL 2156-7085 10.1364/BOE.1.000324 21258470 PMC3005169

[13] Sato T. et al., “Reduction of global interference of scalp-hemodynamics in functional near-infrared spectroscopy using short distance probes,” Neuroimage 141, 120–132 (2016). NEIMEF 1053-8119 10.1016/j.neuroimage.2016.06.054 27374729

[14] Kohno S. et al., “Removal of the skin blood flow artifact in functional near-infrared spectroscopic imaging data through independent component analysis,” J. Biomed. Opt. 12(6), 062111 (2007). JBOPFO 1083-3668 10.1117/1.2814249 18163814

[15] Bauernfeind G. et al., “Separating heart and brain: on the reduction of physiological noise from multichannel functional near-infrared spectroscopy (fNIRS) signals,” J. Neural Eng. 11(5), 056010 (2014).1741-2560 10.1088/1741-2560/11/5/056010 25111822

[16] Funane T. et al., “Quantitative evaluation of deep and shallow tissue layers’ contribution to fNIRS signal using multi-distance optodes and independent component analysis,” NeuroImage 85, 150–165 (2014). NEIMEF 1053-8119 10.1016/j.neuroimage.2013.02.026 23439443

[17] Diamond S. G. et al., “Physiological system identification with the Kalman filter in diffuse optical tomography,” Lect. Notes Comput. Sci. 3750, 649–656 (2005). 10.1007/11566489_80 16686015

[18] Hu X. S. et al., “Kalman estimator- and general linear model-based on-line brain activation mapping by near-infrared spectroscopy,” Biomed. Eng. Online 9, 82 (2010). 10.1186/1475-925X-9-82 21138595 PMC3020171

[19] Zhang Y. H. et al., “Eigenvector-based spatial filtering for reduction of physiological interference in diffuse optical imaging,” J. Biomed. Opt. 10(1), 011014 (2005). JBOPFO 1083-3668 10.1117/1.1852552 15847580

[20] Zhang X. Noah J. A. Hirsch J., “Separation of the global and local components in functional near-infrared spectroscopy signals using principal component spatial filtering,” Neurophotonics 3(1), 015004 (2016). 10.1117/1.NPh.3.1.015004 26866047 PMC4742567

[21] Goodwin J. R. Gaudet C. R. Berger A. J., “Short-channel functional near-infrared spectroscopy regressions improve when source-detector separation is reduced,” Neurophotonics 1(1), 015002 (2014). 10.1117/1.NPh.1.1.015002 26157972 PMC4478749

[r22] Gagnon L. et al., “Short separation channel location impacts the performance of short channel regression in NIRS,” Neuroimage 59(3), 2518–2528 (2012). NEIMEF 1053-8119 10.1016/j.neuroimage.2011.08.095 21945793 PMC3254723

[r23] Saager R. Berger A., “Measurement of layer-like hemodynamic trends in scalp and cortex: implications for physiological baseline suppression in functional near-infrared spectroscopy,” J. Biomed. Opt. 13(3), 034017 (2008). JBOPFO 1083-3668 10.1117/1.2940587 18601562

[24] Takahashi T. et al., “Influence of skin blood flow on near-infrared spectroscopy signals measured on the forehead during a verbal fluency task,” Neuroimage 57(3), 991–1002 (2011). NEIMEF 1053-8119 10.1016/j.neuroimage.2011.05.012 21600294

[25] Gratton G. Corballis P. M., “Removing the heart from the brain: compensation for the pulse artifact in the photon migration signal,” Psychophysiology 32(3), 292–299 (1995). PSPHAF 0048-5772 10.1111/j.1469-8986.1995.tb02958.x 7784538

[26] Ye J. C. et al., “NIRS-SPM: statistical parametric mapping for near-infrared spectroscopy,” Neuroimage 44(2), 428–447 (2009). NEIMEF 1053-8119 10.1016/j.neuroimage.2008.08.036 18848897

[27] Holper L. Scholkmann F. Seifritz E., “Time-frequency dynamics of the sum of intra- and extracerebral hemodynamic functional connectivity during resting-state and respiratory challenges assessed by multimodal functional near-infrared spectroscopy,” Neuroimage 120, 481–492 (2015). NEIMEF 1053-8119 10.1016/j.neuroimage.2015.07.021 26169319

[28] Pinti P. et al., “Current status and issues regarding pre-processing of fNIRS neuroimaging data: an investigation of diverse signal filtering methods within a general linear model framework,” Front. Hum. Neurosci. 12, 505 (2019). 10.3389/fnhum.2018.00505 30687038 PMC6336925

[29] von Luhmann A. et al., “Using the general linear model to improve performance in fNIRS single trial analysis and classification: a perspective,” Front. Hum. Neurosci. 14, 30 (2020). 10.3389/fnhum.2020.00030 32132909 PMC7040364

[30] Shirvan R. A. Setarehdan S. K. Nasrabadi A. M., “A new approach to estimating the evoked hemodynamic response applied to dual channel functional near infrared spectroscopy,” Comput. Biol. Med. 84, 9–19 (2017). CBMDAW 0010-4825 10.1016/j.compbiomed.2017.03.010 28324790

[31] Huppert T. J. et al., “HomER: a review of time-series analysis methods for near-infrared spectroscopy of the brain,” Appl. Opt. 48(10), D280–D298 (2009). APOPAI 0003-6935 10.1364/AO.48.00D280 19340120 PMC2761652

[32] Scholkmann F. et al., “A review on continuous wave functional near-infrared spectroscopy and imaging instrumentation and methodology,” NeuroImage 85, 6–27 (2014). NEIMEF 1053-8119 10.1016/j.neuroimage.2013.05.004 23684868

[33] Brigadoi S. Cooper R. J., “How short is short? Optimum source-detector distance for short-separation channels in functional near-infrared spectroscopy,” Neurophotonics 2(2), 025005 (2015). 10.1117/1.NPh.2.2.025005 26158009 PMC4478880

[34] Gregg N. M. et al., “Brain specificity of diffuse optical imaging: improvements from superficial signal regression and tomography,” Front. Neuroenergetics 2, 14 (2010). 10.3389/fnene.2010.00014 20725524 PMC2914577

[35] Saager R. B. Berger A. J., “Direct characterization and removal of interfering absorption trends in two-layer turbid media,” J. Opt. Soc. Am. A 22(9), 1874–1882 (2005). 10.1364/JOSAA.22.001874 16211814

[36] Diamond S. G. et al., “Dynamic physiological modeling for functional diffuse optical tomography,” Neuroimage 30(1), 88–101 (2006). NEIMEF 1053-8119 10.1016/j.neuroimage.2005.09.016 16242967 PMC2670202

[37] Abdelnour F. Genovese C. Huppert T., “Hierarchical Bayesian regularization of reconstructions for diffuse optical tomography using multiple priors,” Biomed. Opt. Express 1(4), 1084–1103 (2010). BOEICL 2156-7085 10.1364/BOE.1.001084 21258532 PMC3018091

[38] Abdelnour F. Schmidt B. Huppert T. J., “Topographic localization of brain activation in diffuse optical imaging using spherical wavelets,” Phys. Med. Biol. 54(20), 6383–6413 (2009). PHMBA7 0031-9155 10.1088/0031-9155/54/20/023 19809125 PMC2806654

[39] Santosa H. et al., “Investigation of the sensitivity-specificity of canonical- and deconvolution-based linear models in evoked functional near-infrared spectroscopy,” Neurophotonics 6(2), 025009 (2019). 10.1117/1.NPh.6.2.025009 31172019 PMC6541797

[40] Dale A. M., “Optimal experimental design for event-related fMRI,” Hum. Brain Mapp. 8(2–3), 109–114 (1999). HBRME7 1065-9471 10.1002/(SICI)1097-0193(1999)8:2/3<109::AID-HBM7>3.0.CO;2-W 10524601 PMC6873302

[41] Perlman S. B. Huppert T. J. Luna B., “Functional near-infrared spectroscopy evidence for development of prefrontal engagement in working memory in early through middle childhood,” Cereb. Cortex 26(6), 2790–2799 (2016). 53OPAV 1047-3211 10.1093/cercor/bhv139 26115660 PMC4869813

[42] Santosa H. et al., “The NIRS brain analyzIR toolbox,” Algorithms 11(5), 73 (2018).1748-7188 10.3390/a11050073 PMC11218834 38957522

[43] https://bitbucket.org/huppertt/nirs-toolbox.

[44] Pollonini L. Bortfeld H. Oghalai J. S., “PHOEBE: a method for real time mapping of optodes-scalp coupling in functional near-infrared spectroscopy,” Biomed. Opt. Express 7(12), 5104–5119 (2016). BOEICL 2156-7085 10.1364/BOE.7.005104 28018728 PMC5175555

[45] DeLong E. R. DeLong D. M. Clarke-Pearson D. L., “Comparing the areas under two or more correlated receiver operating characteristic curves: a nonparametric approach,” Biometrics 44(3), 837–845 (1988). BIOMB6 0006-341X 10.2307/2531595 3203132

[46] Ma H. et al., “On use of partial area under the ROC curve for evaluation of diagnostic performance,” Stat. Med. 32(20), 3449–3458 (2013). SMEDDA 1097-0258 10.1002/sim.5777 23508757 PMC3744586

[47] Efron B. Tibshirani R. J., An Introduction to the Bootstrap, CRC Press (1994).

[48] Gönen M., “Analyzing receiver operating characteristic curves with SAS,” SAS Institute (2007).

[49] Santosa H. et al., “Characterization and correction of the false-discovery rates in resting state connectivity using functional near-infrared spectroscopy,” J. Biomed. Opt. 22(5), 055002 (2017). JBOPFO 1083-3668 10.1117/1.JBO.22.5.055002 PMC5424771 28492852

