# Supplementary material for: Quantitative comparison of correction techniques for removing systemic physiological signal in functional near-infrared spectroscopy studies
Source: Neurophotonics. 2020 Sep 23;7(3):035009. doi: 10.1117/1.NPh.7.3.035009 (PMC7511246; doi:10.1117/1.NPh.7.3.035009)
Supplement: Supplementary file 1 [file NPh_007_035009_SD001.docx]

**Supplemental**

In this study, subjects preformed a total of 4 types of tasks: resting, breath-hold, walking, and imagined walking. The task sessions all consisted of a 25-second task period followed by a 30-second rest period, which was repeated 5 times. The initial 30-seconds rest period was instructed for restoring values to baseline. For the walking task subjects were instructed to walk at the pace of the treadmill (5km per hour). After the walking task, subjects were instructed to stand still but imagine walking. The breath-hold and resting data were used in the simulations in the main text.

The data for the walking and imagined walking was analyzed using the Brain AnalysIR toolbox in Matlab (www.bitbucket.org/huppertt/nirs-toolbox). Both ordinary least-squares (OLS) and the auto-regressive iterative robust least-squares (AR-IRLS) models were used for comparison. For both of these models, the regression was performed with and without the addition of the short-channel measurements as regressors of no interest in the GLM. These methods have all been detailed in the main text. A group level model was then used to combine the first-level statistical models using a weighted regression model accounting for the first-level noise covariance.

In both the walking and imagined walking, we failed to find any statistically significant activation using analysis procedures. Suppl. Fig. 1 shows the HbO_2_ activation maps of group-level (*t*-value) at *p*<0.05 for walking task. It compares the 4 different processing pipelines without pre-processing: (a) OLS only, (b) OLS with all SS channels as the regression for solving GLM, (c) AR-IRLS only, and (d) AR-IRLS with all SS channels as the regression for solving GLM. Contrary to expectations, this study did not find a significant result for walking task activation. Similarly, suppl. Fig. 2 shows the HbO_2_ activation maps of group-level (*t*-value) at *p*<0.05 for imagine walking task. Here also, we were unable to find the significant result for imagine walking task. Although we did not find significant results, power estimates for the fNIRS channels were beta=0.70 (average) [range 0.31-0.93] and so while the sample size of the study was smaller (5 trials x 2 scans x 12 subjects per condition), we expected to see results given the low level of noise seen in the data. Of note, the statistical power of fNIRS measurements varies widely across channels based on their location on the head, hair thickness, and optode coupling. We feel that these null results could be due to the depth of motor region (i.e., trunk, hip, leg, toes) down the sagittal sulcus.

Suppl. Fig. 1. Activation maps of HbO_2_ for Walking task using OLS (a), OLS with SS-all (b), AR-IRLS (c), and AR-IRLS with SS-all (d) at *q*<0.05.

Suppl. Fig. 2. Activation maps of HbO_2_ for Imagine Walking task using OLS (a), OLS with SS-all (b), AR-IRLS (c), and AR-IRLS with SS-all (d) at *q*<0.05.

“Activation” maps of breath holding task

Breath-holding task will increase blood flow and blood volume throughout the whole brain and this is why was chosen as the experimental controllable physiological noise. We argue this represent an extreme worst-case scenario for a physiological change co-occurring with the task. We expect to see a global response or activations throughout all channels. Suppl. Fig. 3 shows the HbO_2_ *t*-values from all fNIRS files (*x*-axis) and all channels (*y*-axis) for breath-holding task. It also compares 4 different processing pipelines without pre-processing: (a) OLS only, (b) OLS with all SS channels as the regression for solving GLM, (c) AR-IRLS only, and (d) AR-IRLS with all SS channels as the regression for solving GLM. In the plots, the color indicates statistically significant channels whereas black voxels are *p*>0.05. As we expected, the breath-holding task produced very global responses in most subjects, particular with the OLS models (Fig 3 a/b). The AR-IRLS models cut down considerably on the false-discovery due to the breath-hold task even in the case where short-separation regressors were not used (Fig 3c). Short-separation (SS) channels were used in the regression and result in some reduction the global response (see panels a and d). This was mode pronounced for the OLS model than the AR-IRLS. Overall the AR-IRLS performed better to reduce those global response (panels c,d compared with a, b).

za

Suppl. Fig. 3. T-statistic values of HbO_2_ from all fNIRS files (*x*-axis) and all channels (*y*-axis) for breath-holding task using OLS (a), OLS with SS-all (b), AR-IRLS (c), and AR-IRLS with SS-all (d). Black color means -*p*<0.05 (|T-statistic|<1.96).

Nearest SS channels as a pre-filter and regressor

Suppl. Fig. 4 displays the comparison of AUC values of the sensitivity-specificity reports from various SS channel as a pre-filter (panel a) and SS channel as a regression for solving GLM (panel b). Those figures also compare the performance using the nearest SS channels from 1- up to 8-channel. In every panel, it shows the result from 3 datasets (resting: blue, BH (random): green, BH-locked: red) and sub-type of pipelines (HbO_2_ only: solid-line and HbO_2_&Hb: dotted-line). Similar with the previous findings in Fig. 3 of the main text, (i) it is better to use the SS-GLM compared to using the SS-filter, (ii) combining HbO_2_&Hb for both SS-filter and SS-GLM have slightly better performance than HbO_2_ only. It is noted that the right figure (panel b) is similar with Fig. 6 of the main text. In addition, by increasing the number of SS channels, the performance has slightly increased in AUC.

 Suppl. Fig. 4. Comparison of sensitivity-specificity of AUC using various nearest SS channels for 3 datasets (resting, BH-random, BH-locked) and 2 sub-type of pipelines (HbO_2_ only and HbO_2_&Hb). All these nearest SS channels have been implemented as a pre-filter (a) and SS as a regression for solving GLM (b).

Examination of the number of needed short-separation measurements

In Fig. 5 of the main text, we showed that the area-under-the-curve in the ROC analysis increased up to the maximum number (8) of short-separation channels that were recorded in the probe. This means that information from the furthest short separation measurement is still improving the models for data on the opposite side of the probe. In this analysis, a specific GLM was set up for each individual channel. In order to examine if this was a general finding across all subjects, suppl. Fig. 5 shows the percentage of the variance data by using singular value decomposition of all 8 short separation channels for all the subjects. We processed all the files from 12 subjects and 7 sessions for both HbO_2_ (upper) and Hb (lower). The thick black solid-line represents the averaged over all the files and thin red dotted-line is the 90% threshold. The figure shows that about 5 components model 90% of the variance in the hemoglobin data (HbO_2_ and Hb). The first component (a single global regressor) only models about 40-50% of the variance. Thus, a single-short distance measurement would not be sufficient for the bilateral motor-cortex probe used in this study. On the other hand, the plateau of the eigenvalues around 5 components suggests that there a fair amount of spatial structure to these physiological signals and that it may not be much benefit going too much above the 8 positions used in this study. It is *unclear* how this result would generalize outside of the areas of the brain used in this study.


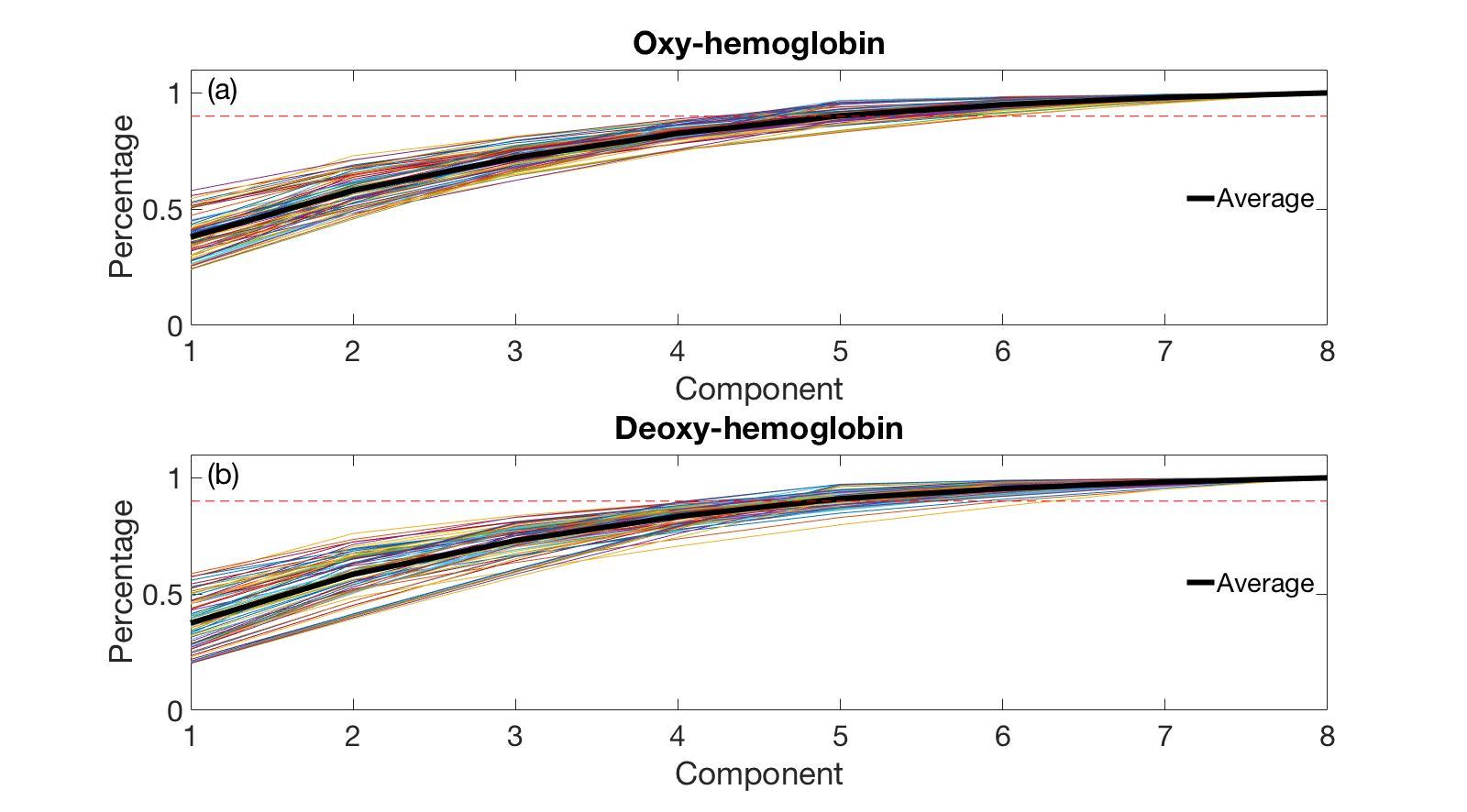


Suppl. Fig. 5. Percentage variance of oxy-hemoglobin (a) and deoxy-hemoglobin (b) of short-separation channels using 1 component up to all components. Singular value decomposition has been applied to every files data including resting, walking, imagine walking, and breath-holding task. Thicker black line is the average from all files.
